# Opioids Alleviate Oxidative Stress via the Nrf2/HO-1 Pathway in LPS-Stimulated Microglia

**DOI:** 10.3390/ijms241311089

**Published:** 2023-07-04

**Authors:** Akash Shivling Mali, Ondrej Honc, Lucie Hejnova, Jiri Novotny

**Affiliations:** Department of Physiology, Faculty of Science, Charles University, 12800 Prague, Czech Republic; akash.mali@natur.cuni.cz (A.S.M.); ondrej.honc@natur.cuni.cz (O.H.); lucie.hejnova@natur.cuni.cz (L.H.)

**Keywords:** microglia, opioids, lipopolysaccharide, oxidative stress, glucose transporter, NADPH, Nrf2/HO-1

## Abstract

Opioids are known to have antioxidant effects and to modulate microglial function under certain conditions. It has been previously shown that opioid ligands can effectively inhibit the release of proinflammatory cytokines when stimulated with lipopolysaccharide (LPS) and convert microglia to an anti-inflammatory polarization state. Here, we used C8-B4 cells, the mouse microglial cell line activated by LPS as a model to investigate the anti-inflammatory/antioxidant potential of selected opioid receptor agonists (DAMGO, DADLE, and U-50488). We found that all of these ligands could exert cytoprotective effects through the mechanism affecting LPS-induced ROS production, NADPH synthesis, and glucose uptake. Interestingly, opioids elevated the level of reduced glutathione, increased ATP content, and enhanced mitochondrial respiration in microglial cells exposed to LPS. These beneficial effects were associated with the upregulation of the Nrf2/HO-1 pathway. The present results indicate that activation of opioid signaling supports the preservation of mitochondrial function with concomitant elimination of ROS in microglia and suggest that an Nrf2/HO-1 signaling pathway-dependent mechanism is involved in the antioxidant efficacy of opioids. Opioid receptor agonists may therefore be considered as agents to suppress oxidative stress and inflammatory responses of microglia.

## 1. Introduction

It has long been recognized that different neurological and neurodegenerative diseases, including Alzheimer’s disease (AD), Parkinson’s disease (PD), and multiple sclerosis (MS), are associated with the pathophysiological process of neuroinflammation [1,2,3]. Neuroinflammatory responses caused by pathogenic factors, including excessive production of reactive oxygen species (ROS), increased NADPH oxidase activity, glutathione redox imbalance, and secretion of proinflammatory cytokines such as tumor necrosis factor (TNF)-α, interleukin (IL)-1, IL-6, IL-12 and CD 86, can be detrimental to healthy neurons, and lead to neuronal death, synaptic dysfunction, and loss of synapses [4]. Suppression of inflammatory responses might be beneficial to prevent the progression of neurodegenerative diseases. Microglia represent the first line of defense in the CNS [5,6], and they have been well-known for a long time as key players in many neurodevelopmental, neurological, and neurodegenerative conditions. Stimulation with inflammatory stimuli, such as lipopolysaccharide (LPS), can induce microglia to exacerbate inflammation by over-releasing proinflammatory factors that cause neuronal dysfunction and cell death [7]. Growing evidence indicates that ROS are secondary messengers in microglia which promote progressive inflammatory processes and may contribute to immune dysregulation [8,9].

A key role in maintaining cellular redox balance, optimal ATP production, and mitochondrial function is attributed to complex antioxidant defense systems. One of the major regulators of cellular resistance to oxidants is the nuclear transcription factor erythroid 2-related factor-2 (Nrf2), which controls the expression of several antioxidant enzymes [10]. Recent evidence suggests that key cell modulators such as DJ-1, VEGF, oxLDL, LXA4, AOPPs, miR-133a-3p, CD151, and BRD4 have the ability to regulate Nrf2, thereby enhancing the cellular response to oxidative stress and preventing cell death in placental cells, prostate cancer, cervical cancer, and endometrial cancer [11,12]. The use of Nrf2 activators may promote and prevent the progression of advanced stages of MAFLD, such as liver fibrosis and cirrhosis [13]. Nrf2 has recently been described as an important factor for immunomodulatory and anti-inflammatory properties through the induction of heme oxygenase-1 [14,15,16]. Recent studies have shown that HO-1 is triggered in most tissues by various oxidative stimuli and plays a protective role against various forms of tissue damage and cellular injury caused by oxidants. It has also been demonstrated that HO-1 has significant immunomodulatory and anti-inflammatory functions. 

Emerging research suggests that metabolic reprogramming plays an important role in regulating the innate inflammatory response. Alteration of metabolic functions or transition from a growth-promoting state to a disease-associated microglial state allows microglia to perform their functions effectively in different contexts [17,18,19]. Under normal conditions of oxygenation, cells obtain their energy through two distinct mechanisms. The first involves the conversion of glucose to pyruvate via glycolysis, which then enters the mitochondrial tricarboxylic acid cycle to generate ATP through oxidative phosphorylation. However, under hypoxic conditions, pyruvate is converted to lactate by anaerobic glycolysis. Importantly, immune cells have the ability to switch from oxidative phosphorylation to aerobic glycolysis, which is similar to the Warburg effect observed in tumor cells [20]. In this transition, cells give preference to glycolysis over catabolic mitochondrial pathways to maintain and generate the metabolic resources required for cell proliferation and activation while still ensuring an adequate ATP supply.

There is some evidence that opioids have antioxidant effects and may modulate microglial function. Opioid ligands transmit their signals through opioid receptors (ORs), which are widely distributed in the central nervous system [21]. We have previously reported that ORs (μ-, δ-, and κ-ORs) are present in microglia, and we observed that their activation could convert the LPS-induced proinflammatory subset of microglia into an anti-inflammatory subset by inhibiting the excessive production of nitric oxide, decreasing the expression of proinflammatory cytokines, and increasing the expression of anti-inflammatory molecules [22]. There is growing evidence that opioids may be neuroprotective under certain conditions [23,24,25,26]. In the present study, we investigated the mechanism underlying the antioxidant and anti-inflammatory effects of selected OR agonists (DAMGO, DADLE, and U-50488) in microglial cells. As a model, we used the C8-B4 mouse microglial cell line and activation with the bacterial endotoxin LPS. We also examined the effects of LPS and opioids on mitochondrial respiration and ATP production. Our results suggest that the deleterious effects of LPS in microglia can be abrogated by opioid receptor agonists, and we show for the first time that the Nrf2/HO-1 pathway may be involved in these beneficial effects.

## 2. Results

### 2.1. Opioid Receptor Agonists Do Not Affect Microglial Viability and Reduce LPS Cytotoxicity

**Figure 1:**
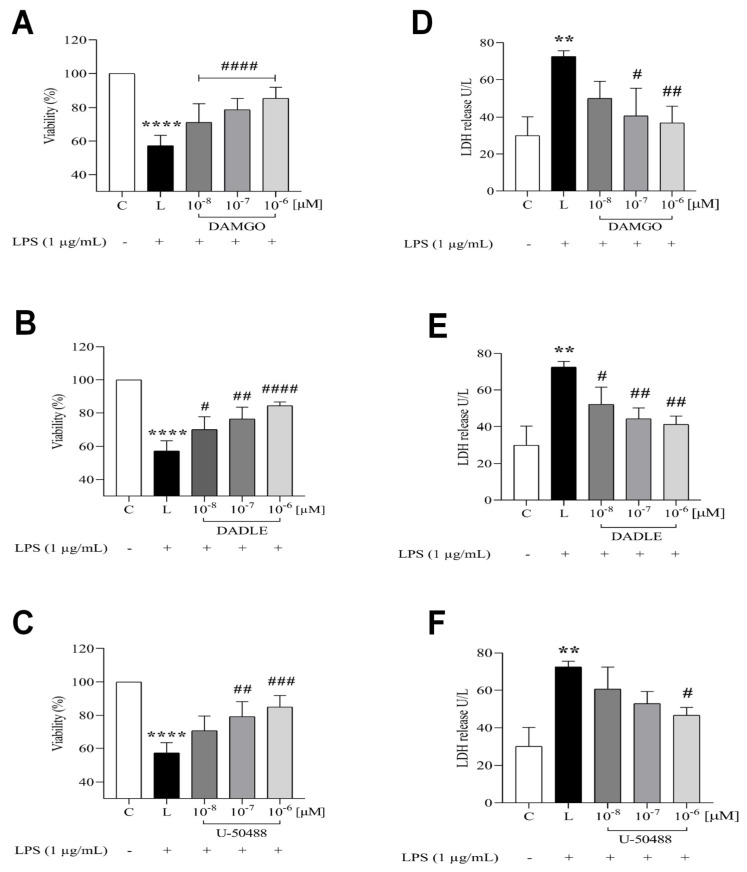
Effects of LPS and opioid receptor agonists on cell viability and LDH release. C8-B4 cells were either untreated (control, C) or pretreated with different concentrations (0.01–1 µM) of DAMGO (**A**,**D**), DADLE (**B**,**E**), and U-50488 (**C**,**F**) for 1 h and then incubated in the presence of LPS (1 µg/mL, L) for 24 h. Cell viability was determined using the MTT assay, and cytotoxicity of LPS was determined using the Cytotoxicity Detection KitPLUS LDH (Merck). Data are presented as means ± SEM of three independent experiments performed in triplicate (n = 3) (** *p* < 0.01, **** *p* < 0.0001 compared with the control; ^#^ *p* < 0.05, ^##^ *p* < 0.01, ^###^ *p* < 0.001, ^####^ *p* < 0.0001, compared with LPS-treated group).

After treatment of C8-B4 cells with increasing concentrations (from 0.01 to 1 μM) of selected opioid ligands (DAMGO, DADLE, and U-50488) for 24 h, cells were incubated for an additional 24 h in the absence or presence of (1 μg/mL). Then, cell viability and drug toxicity were measured by MTT assay and LDH release assay, respectively. Opioid ligand treatment alone had no effect on cell viability and no cytotoxic effects (Supplementary Figure S1A). Opioid receptor agonists at concentrations of 0.01 μM or higher were used for all further studies. In contrast, treatment of cells with LPS resulted in a decrease in cell viability of approximately 50% after 24 h. Pretreatment with DAMGO, DADLE, or U-50488 dose-dependently improved cell viability (Figure 1A–C) and reduced the cytotoxicity of LPS (Figure 1D–F). Hence, the cytotoxicity of LPS can apparently be attenuated by pretreatment of cells with increasing concentrations of opioid receptor agonists.

### 2.2. Opioid Receptor Agonists Prevent the LPS-Induced Increase in Glucose Uptake and Downregulate GLUTs

**Figure 2:**
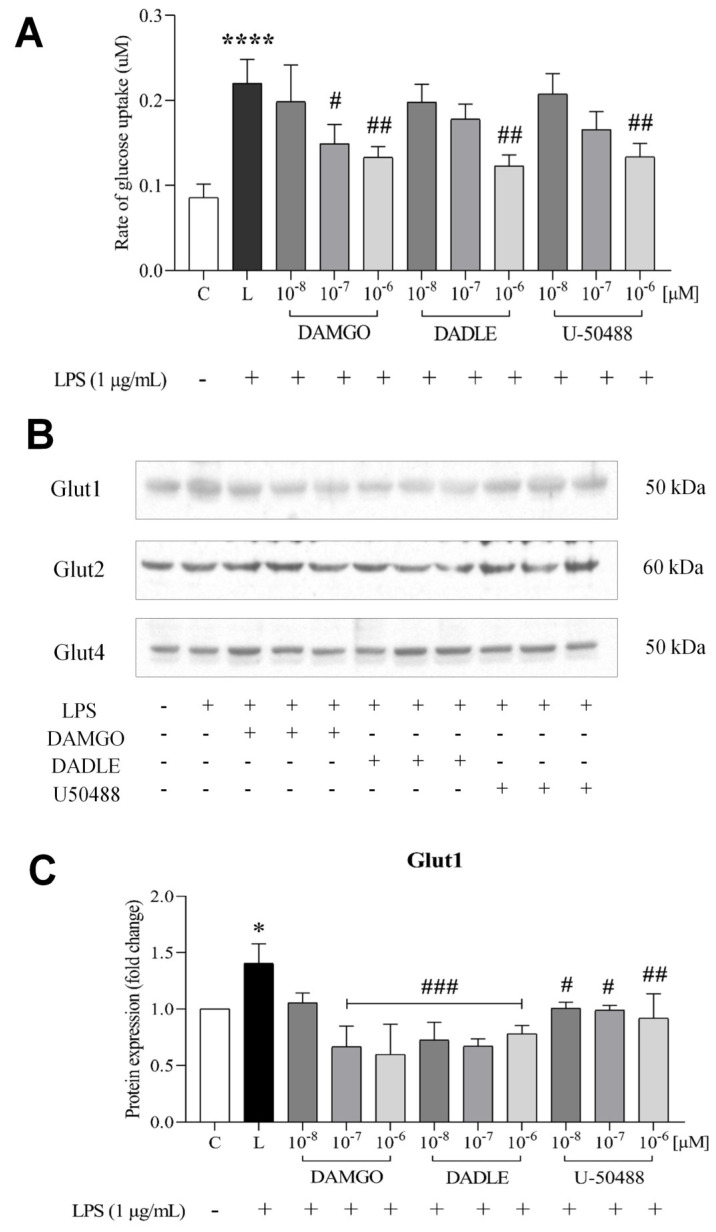
Effect of LPS and opioid ligands on glucose uptake and GLUTs expression. C8-B4 cells were either untreated (control, C) or pretreated with different concentrations (0.01–1 µM) of DAMGO, DADLE, and U-50488 for 1 h and then incubated in the presence of LPS (1 µg/mL, L) for 24 h. Glucose uptake (**A**) was measured using the Glucose Uptake-Glo Assay (Promega). Expression of GLUTs was determined by Western blot analysis (**B**), and relative GLUT1 levels were expressed as fold changes compared with control (**C**). Values represent the mean ± SEM of three independent experiments (n = 3) (* *p* < 0.05, **** *p* < 0.0001, compared with the control; ^#^ *p* < 0.05, ^##^ *p* < 0.01, ^###^ *p* < 0.001 compared with LPS-treated group).

Some previous studies suggest that glucose may contribute to inflammatory events in microglial cells [27,28,29]. Here, we investigated whether selected opioid ligands can affect the rate of glucose transport in LPS-stimulated microglial cells. To this end, we measured the uptake of 2-deoxyglucose (2-DG). We observed that treatment of cells with LPS led to a 2-fold increase in the uptake of 2-DG (Figure 2A). Interestingly, the pretreatment of cells with opioids suppressed the uptake of 2-DG in a concentration-dependent manner. Specifically, LPS-stimulated uptake of 2-DG decreased by approximately 40% in cells pretreated with 1 μM DAMGO, DADLE, or U-50488 (Figure 2A). We also wanted to determine which glucose transporters (GLUTs) are involved in glucose uptake in C8-B4 microglia. Therefore, we examined the expression of different GLUTs in C8-B4 microglia by Western blot analysis (Figure 2B), which revealed the presence of GLUT1, 2, and 4 but not GLUT3 in these cells. To describe the relationship between glucose metabolism and microglial activation, we stimulated microglial cells with LPS and OR agonists and LPS and then determined the expression levels of GLUTs. We found that GLUT1 increased significantly in LPS-treated cells compared with control cells, whereas GLUT2 and GLUT4 were unaffected by LPS or opioid treatment (Supplementary Figure S1H,I). We also found that DAMGO (0.1 and 1 μM) and DADLE and U-50488 (0.01–1 μM) downregulated LPS-induced GLUT1 expression (Figure 2C).

### 2.3. Opioid Receptor Agonists Inhibit the LPS-Induced Inflammatory Response by Increasing Antioxidant Capacity, Preventing the Increase in NADPH Production, and Promoting GSH Upregulation

**Figure 3:**
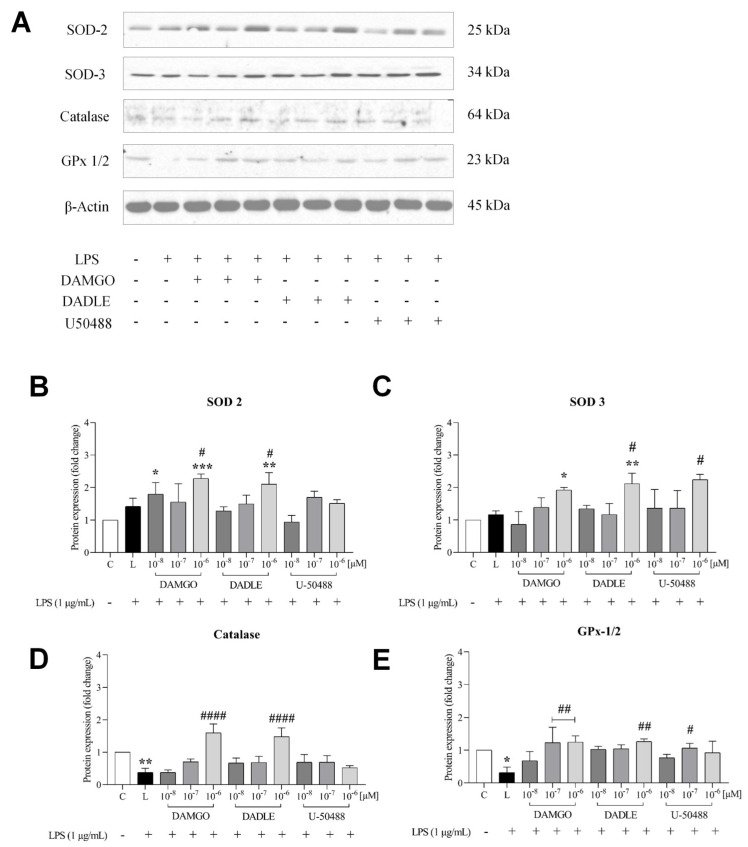
Effect of LPS and opioid receptor agonists on the expression of selected antioxidant markers. C8-B4 cells were either untreated (control, C) or pretreated with opioid ligands (DAMGO, DADLE, U-50488) at a concentration of 0.01–1 µM for 1 h and then incubated in the presence of LPS (1 μg/mL, L) for 24 h. The levels of selected antioxidant enzymes were determined by Western blot analysis (**A**), and the relative levels of SOD2 (**B**), SOD3 (**C**), catalase (**E**), and GPx 1/2 (**D**) were expressed as fold changes compared with the control. Values represent the mean ± SEM of three independent experiments (n = 3) (* *p* < 0.05, ** *p* < 0.01, *** *p* < 0.001, compared with control; ^#^ *p* < 0.05, ^##^ *p* < 0.01, ^####^ *p* < 0.0001 compared with LPS-treated group).

Because chronic inflammation may be associated with oxidative stress, we examined the antioxidant activity of opioid ligands and monitored protein expression of antioxidant enzymes such as superoxide dismutase (SOD2, SOD3), catalase (CAT), and glutathione peroxidase (GPX 1/2) by Western blot analysis (Figure 3). We observed that selected opioids attenuated the accumulation of intracellular ROS and superoxide anions (O_2_^•−^) associated with inflammation. Pretreatment with opioid receptor ligands at a concentration of 1 μM significantly increased the level of SOD, catalase, and glutathione peroxidase (Figure 3B–E). The expression of these enzymes was not significantly affected by opioids alone (Supplementary Figure S2).

**Figure 4:**
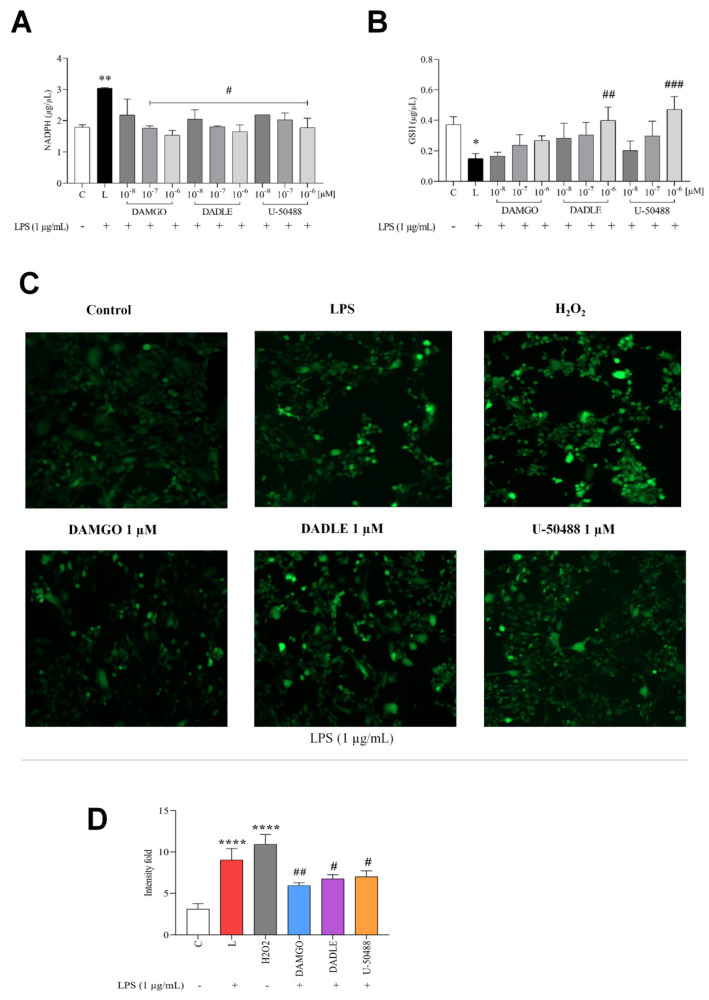
Effect of LPS and opioid ligands and LPS on NADPH and GSH levels and intracellular ROS production. C8-B4 cells were either untreated (control, C) or pretreated with opioid ligands (DAMGO, DADLE, U-50488) at various concentrations for 1 h and then incubated in the presence of LPS (1 μg/mL, L) for 24 h. When ROS were evaluated, cells were treated with H_2_O_2_ (50 μM) as a positive control. The levels of NADPH (**A**) and GSH (**B**) were determined using appropriate kits as described in Methods. DCFDA staining was used to estimate intracellular ROS (**C**), and the fluorescence intensity of individual preparations was quantitatively evaluated using ImageJ (**D**). Values represent the mean ± SEM of three independent experiments (n = 3) (* *p* < 0.05, ** *p* < 0.01, **** *p* < 0.001 compared with control; ^#^ *p* < 0.05, ^##^ *p* < 0.01, ^###^ *p* < 0.001 compared with LPS-treated group).

Because NADPH is required for the production of ROS by NADPH oxidase and some glucose is metabolized via the pentose monophosphate shunt to generate reducing equivalents for the regeneration of NADPH from NADP^+^, we examined here whether intracellular NADPH levels were affected by LPS and OR agonists. We found that NADPH synthesis increased by more than 40% after treatment of microglia with LPS (Figure 4A), indicating that LPS promoted NADPH production. On the other hand, NADPH production was reduced to control levels and even further in the presence of all three opioids tested (Figure 4A).

Opioids are known to have antioxidant effects under certain conditions [30]. Therefore. Therefore, we decided to investigate the effects of opioid ligands on the level of the antioxidant glutathione (GSH) in LPS-stimulated C8-B4 microglia. As shown in Figure 4B, GSH levels decreased by approximately 60% in PLS-treated cells, and in contrast to DAMGO, DADLE, and U-50488 were able to reverse this effect at the highest (1 μM) concentrations.

### 2.4. Opioid Receptor Agonists Reverse LPS-Induced Dysregulation of Cellular Redox Balance and Bioenergetics

**Figure 5:**
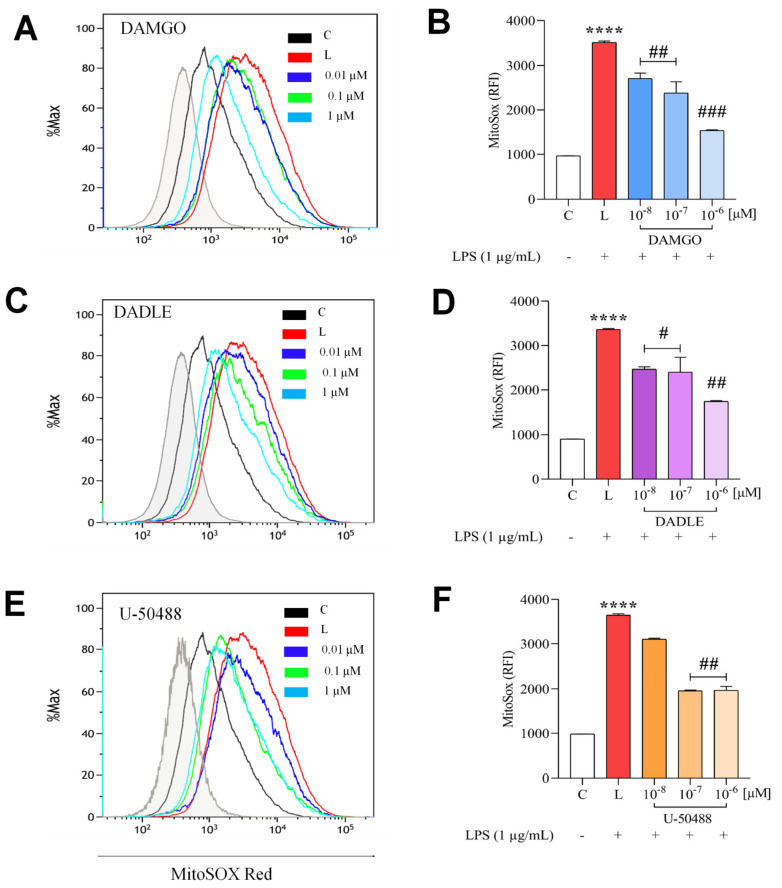
Effect of LPS and opioid ligands on mitochondrial ROS. C8-B4 cells were either untreated (control, C) or pretreated with opioid ligands (DAMGO, DADLE, U-50488) at various concentrations for 1 h and then incubated in the presence of LPS (1 μg/mL, L) for 24 h. Mitochondria ROS were assessed by flow cytometric analysis using MitoSOX red probe. Representative flow cytometric histograms (**left panels**) and analyses (**right panels**) of each experimental group: DAMGO (**A**,**B**), DADLE (**C**,**D**), and U-50488 (**E**,**F**). Values represent the median ± SEM from 3 independent experiments (n = 3) (**** *p* < 0.0001 compared with control; ^#^ *p* < 0.05, ^##^ *p* < 0.01, ^###^ *p* < 0.001 compared with LPS-treated group).

We investigated the effect of LPS and opioid receptor ligands on cellular redox balance in C8-B4 cells. H2DCF-DA, a cell-permeable fluorescent probe, was used to measure intracellular ROS in C8-B4 cells (Figure 4C,D). As expected, the baseline value of intracellular ROS was low in control (unstimulated) cells, and there was a significant increase after stimulation of cells with 1 μg/mL LPS or 50 μM hydrogen peroxide (Figure 4C). Pretreatment of cells with selected opioid ligands significantly suppressed LPS-induced ROS production (Figure 5). DCF fluorescence detects total intracellular ROS and does not specify the source. Mitochondrial ROS were analyzed using MitoSOX Red, a more specific fluorescence probe that targets mitochondria, where it is oxidized by mitochondrial ROS. Flow cytometry analysis with MitoSOX Red staining showed that treatment of cells with LPS resulted in a significant increase (by about 3.5-fold) in MitoSOX Red fluorescence intensity, indicating strong ROS formation. Importantly, the deleterious effect of LPS was attenuated by the pretreatment of cells with DAMGO, DADLE, or U-50488 in a dose-dependent manner. Specifically, LPS-induced ROS production was reduced 1- and 2-fold in the presence of 0.01–1 μM DAMGO (Figure 5B) or DADLE (Figure 5D), respectively, and 2-fold in the presence of 0.1 and 1 μM U-50488 (Figure 5F).

**Figure 6:**
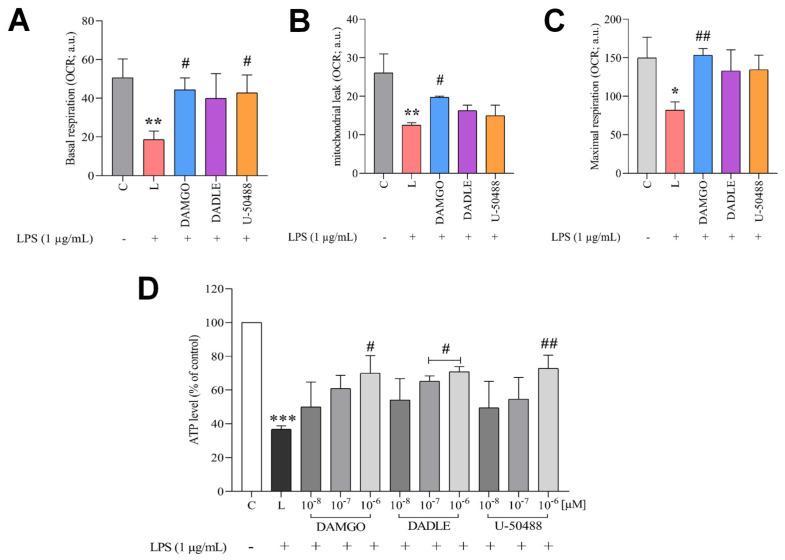
Effect of LPS and opioid receptor ligands on mitochondrial respiration and ATP content. C8-B4 cells were either untreated (control, C) or pretreated with opioid ligands (DAMGO, DADLE, U-50488) at a concentration of 1 µM for 1 h and then incubated in the presence of LPS (1 μg/mL, L) for 24 h. Mitochondrial respiration was assessed as extracellular oxygen consumption rate (OCR). OCR was measured under basal conditions (**A**) followed by sequential addition of oligomycin (0.5 µM) to determine mitochondrial leak (**B**), and FCCP (50 nM) and antimycin A (2.5 µM) to determine maximal respiration (**C**). OCR values were normalized to a number of cells. Relative changes in ATP content (**D**) were determined using a bioluminescence kit. Values represent the mean ± SEM of three independent experiments (n = 3) (* *p* < 0.05, ** *p* < 0.01, *** *p* < 0.001 compared with control; ^#^ *p* < 0.05, ^##^ *p* < 0.01 compared with LPS-treated group).

Next, we investigated the effects of LPS and opioids on the functional state of mitochondrial respiration in C8-B4 cells using an OROBOROS Oxygraph-2k (Supplementary Figure S3). Mitochondrial respiration was assessed by real-time measurements of extracellular oxygen consumption under different experimental conditions. Treatment of cells with LPS resulted in a marked decrease in basal and maximal respiration rate (Figure 6A,B). Leak-driven oxygen consumption rate (OCR) also significantly decreased in cells exposed to LPS (Figure 6C). This indicates a significant loss of electron transport chain (ETC) capacity. Pretreatment of cells with DAMGO, DADLE, and U-50488 at a concentration of 1 μM preserved the ETC capacity and almost completely rescued basal mitochondrial respiration.

The effects of LPS and OR agonists on intracellular ATP content were also examined (Figure 6D). The bioluminescence assay for the detection of intracellular ATP content showed a decreased amount of ATP in C8-B4 cells treated with LPS. The LPS-induced decrease in intracellular ATP was significantly reduced by pretreatment of the cells with opioid ligands.

### 2.5. Opioid Receptor Agonists Activate the Nrf2/HO-1 Pathway in LPS-Treated Microglia

**Figure 7:**
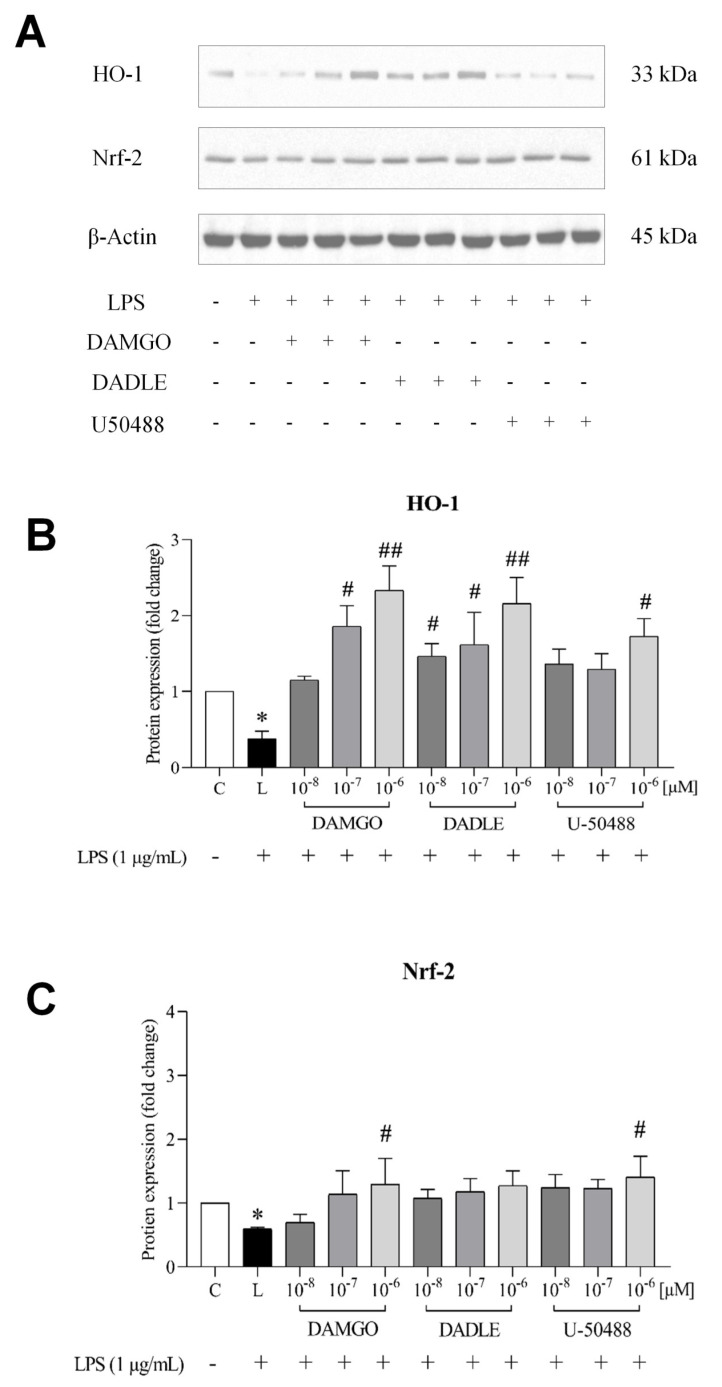
Effect of LPS and opioid receptor agonists on the expression of Nrf2 and HO-1. C8-B4 cells were either untreated (control, C) or pretreated with opioid ligands (DAMGO, DADLE, U-50488) at various concentrations for 1 h and then incubated in the presence of LPS (1 μg/mL, L) for 24 h. Selected components of the Nrf2 pathway were determined by Western blot analysis (**A**). The relative levels of HO-1 (**B**) and Nrf2 (**C**) were expressed as fold changes compared with control. Values represent the mean ± SEM of three independent experiments (n = 3) (* *p* < 0.05 compared with control; ^#^ *p* < 0.05, ^##^ *p* < 0.01 compared with LPS-treated group).

A recent study has shown that morphine can activate Nrf2, which in turn leads to increased transcriptional activation of HO-1 expression [31]. Previous research also suggested that morphine-induced anti-inflammatory effects can occur through Nrf2 and HO-1 activation [32]. Here, we examined whether ORs activation by DAMGO, DADLE, and U-50488 may affect the expression of Nrf2 and HO-1. Western blot analysis showed that after treatment with LPS, the expression of Nrf2 and HO-1 was downregulated, whereas pretreatment of cells with selected opioid ligands (DAMGO and U50488) upregulated Nrf2 and especially HO-1 in a dose-dependent manner (Figure 7). Exposure of C4-B4 cells to opioid receptor agonists alone did not significantly affect Nrf2 or HO-1 expression (Supplementary Figure S2).

## 3. Discussion

In this study, we investigated the anti-inflammatory effects of selected μ-, δ-, and κ-OR agonists (DAMGO, DADLE, and U-50488, respectively) in LPS-stimulated C8-B4 microglial cells. We found that all these opioids were able to significantly suppress LPS-induced oxidative stress and that Nrf2/HO-1 signaling was involved in the ameliorative effects that promoted cell viability. This was associated with decreased LDH release, a marker of cell damage, in the presence of opioid receptor agonists, consistent with the previously reported cytoprotective effects of morphine [33,34]. We also observed that treatment of cells with LPS increased glucose uptake and NADPH content and decreased the level of reduced GSH and that these deleterious effects were abolished by opioid receptor agonists pretreatment in a dose-dependent manner. These results suggest that opioids are capable of reducing cytotoxic effects during neuroinflammation.

As previously reported, increased glucose uptake appears to play a key role in microglial activation, particularly under inflammatory conditions, and is associated with metabolic reprogramming of microglia toward anaerobic glycolysis [18,28,35]. Here, we observed that the glucose consumption in C8-B4 cells, which was markedly increased by LPS, was significantly reduced by opioids, suggesting that these ligands can curb the inflammatory response by reducing glucose uptake. To determine how glucose transport might be affected, we examined the expression of selected GLUTs under different experimental conditions. Of the four detected isoforms of GLUT (GLUT1, GLUT2, GLUT3, and GLUT4), only the dominant GLUT1 was strongly altered by treatment with LPS and opioids. The expression of GLUT1 was markedly upregulated by LPS, and this effect was suppressed in the presence of opioid receptor agonists. These results support the notion that microglia increase GLUT1 expression to enhance glucose uptake under inflammatory conditions and that opioids can support cytoprotection by modulating GLUT1 expression. Interestingly, the ability of opioids to affect glucose transport may be cell type-specific, as activation of μ-OR and δ-OR increased glucose uptake in C2C12 myoblasts and Chinese hamster ovary cells, respectively [36]. As stated above, opioids were able to prevent LPS-induced upregulation of GLUT1 and decrease glucose uptake in microglia; however, they did not completely inhibit glucose uptake, even at higher concentrations. This may be due to the incomplete elimination of GLUT1 and the involvement of other GLUTs in glucose uptake by microglia.

Glucose provided by increased glucose uptake in LPS-stimulated microglia could apparently serve, at least part, for the synthesis of nucleotide precursors. In this context, it is worth noting that glucose-6-phosphate can be diverted to the pentose phosphate shunt, which generates nucleotide precursors and regenerates NADPH, an essential substrate for NADPH oxidase [37,38,39]. Importantly, NADPH synthesis was significantly upregulated in LPS-treated cells. In parallel, mitochondrial respiration was significantly lower in LPS-activated microglia, which was associated with markedly decreased intracellular ATP levels. The ability of opioid receptor agonists to reduce LPS-induced suppression of mitochondrial respiration and ATP production and to inhibit LPS-promoted NADPH production could likely contribute to the anti-inflammatory effect of these drugs. Other treatments that prevent LPS-mediated inflammation in microglia also efficiently reduce NADPH synthesis, suggesting that the inhibitory effects of opioids on glucose consumption and NADPH synthesis enhance their intrinsic antioxidant potential in a self-reinforcing process. These effects may be evident at low micromolar concentrations of opioid ligands, which have only moderate antioxidant properties but potent anti-inflammatory effects.

Chronic inflammation is a typical feature in various neurodegenerative diseases, commonly thought to be triggered by oxidative stress. Overproduction of ROS by infiltrating immune cells can be extremely harmful and toxic, acting as potent mediators of brain damage in brain inflammation [40]. LPS is known to induce oxidative stress under various conditions due to excessive production of ROS, including in models of neuroinflammation and neurodegeneration [41,42,43]. There is some evidence that sustained activation of ORs can attenuate the accumulation of intracellular ROS, thereby averting oxidative stress in some experimental models [44]. In the present study, selected opioid receptor agonists were found to effectively reduce intracellular and mitochondrial ROS production in LPS-stimulated C8-B4 cells. Opioid pretreatment of cells before LPS addition significantly reduced the production of superoxide, a potent mediator of brain damage during inflammation, and oxidative damage to cell membranes.

Excessive production of ROS can lead to lipid peroxidation, which is particularly harmful to CNS cells because of the high content of polyunsaturated fatty acids [45]. Lipid hydroperoxides and H_2_O_2_ can be detoxified by glutathione peroxidases, antioxidant enzymes that play an important role in preventing lipid peroxidation [46]. Here, we observed that GPX1/2 expression was significantly downregulated by treatment of C8-B4 microglia with LPS and that opioid receptor agonists prevented this downregulation. In addition, these drugs also increased cellular levels of reduced GSH, a major cellular antioxidant in both neuronal and nonneuronal cells. GSH reacts with ROS to lower their amounts, and helps maintain acceptable levels of oxidative stress [47,48,49]. GSH acts alone or in conjunction with enzymes to reduce superoxide and hydroxyl radicals and peroxynitrites generated during normal cellular metabolism and is a preferred substrate for several enzymes involved in antioxidant protection and xenobiotic metabolism [50,51]. GSH deficiency has been implicated in aging, and a number of diseases, including neurodegeneration, and GSH supplementation has been found to alleviate inflammation [52]. Our results dealing with superoxide dismutase and catalase, the enzymes important for the reduction of superoxide anion radicals (O_2_^−^) to H_2_O_2_ and subsequent conversion to H_2_O and O_2_, indicated that these antioxidant enzymes might also be involved in the protective effects of opioids in microglia because they were upregulated in cells pretreated with these drugs. These observations are consistent with previously reported increases in catalase expression in H9c2 cardiomyoblasts treated with morphine [34] and higher levels of serum SOD and GPX in rabbits treated with DADLE [30].

**Figure 8:**
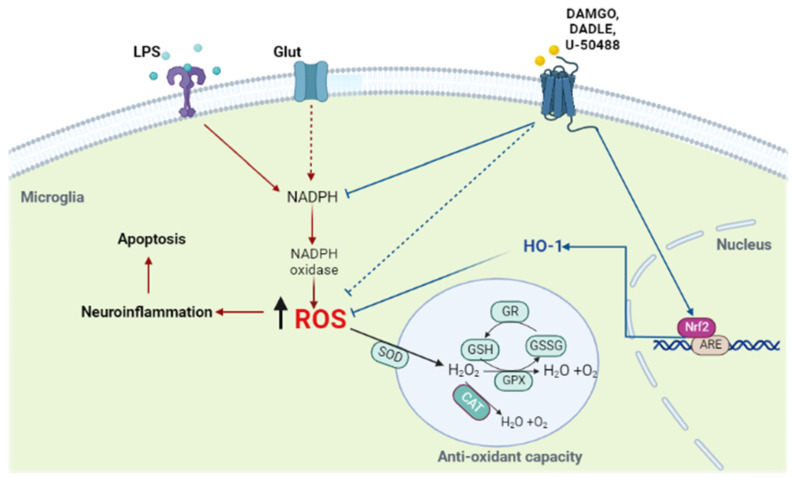
Opioid receptor agonists attenuate LPS-induced neuroinflammation by preventing the production of ROS via the Nrf2/HO-1 pathway—schematic representation of how opioid receptor agonists can reduce LPS-induced microglial inflammation. After entering the nucleus, Nrf2 binds to the antioxidant response element (ARE) and promotes the transcription of a number of antioxidant ARE–dependent genes, including HO-1. As a result, antioxidant capacity increases, intracellular ROS are reduced, and the microglia-mediated inflammatory response could be attenuated. Opioid receptor agonists could prevent microglial apoptosis by maintaining mitochondrial function while blocking excessive glucose uptake, NADPH loss, and ATP depletion.

It is known that the transcription of genes encoding oxidant-neutralizing enzymes, such as SOD2, catalase, and GPX, is regulated by Nrf2 [53,54,55,56]. Normally, Nrf2 is bound to Keap1 in the cytoplasm and keeps it in an inactive state. However, under oxidative stress, Nrf2 dissociates from Keap1 and translocates to the nucleus, where it activates the antioxidant response element (ARE) and increases the expression of Nrf2-regulated genes, including HO-1, another potentially important component of cellular resistance to oxidants [15,16,57,58]. To further explore the mechanism behind opioid-mediated protection against LPS-induced oxidative stress in microglia, we examined the expression of this transcription factor and HO-1. We found that LPS decreased the expression of Nrf-2 and HO-1 and that pretreatment of cells with opioid receptor agonists prevented LPS-induced downregulation of Nrf-2 and significantly enhanced the expression of HO-1. Interestingly, regulation of HO-1 by LPS in macrophages and monocytes has led to partially contradictory results. While treatment of rat Kuppfer cells or human monocytic leukemia THP-1 cells and human monocytes with LPS resulted in a marked upregulation of HO-1 [59,60], no effect of LPS on HO-1 expression was observed in murine J774 macrophages [61]). These seemingly disparate results can be explained by different experimental conditions. It seems that HO-1 can be affected differently by LPS depending on the cell type and specific exposure conditions. Our data are in line with the previously observed activation of the Nrf2/HO-1 pathway by μ-OR agonists in the lung [62,63] or by κ-OR agonists in the heart and brain [64,65] and support the assumption of crucial involvement of this pathway in the inhibition of LPS-induced oxidative stress by opioids in microglia (Figure 8).

In summary, the results of the present study demonstrate that opioid receptor agonists (DAMGO, DADLE, U-50488) exert potent antioxidant and anti-inflammatory effects in LPS-stimulated C8-B4 cells and that the Nrf2/HO-1 signaling pathway is substantially involved in mediating these effects. Furthermore, this is the first study to show the positive effects of activation of ORs on microglial energy metabolism in LPS-induced oxidative stress. These results confirm that modulation of OR-mediated signaling may be potentially useful in preventing and/or treating microglial inflammation, thus providing additional support for the treatment of neurodegenerative diseases.

## 4. Materials and Methods

### 4.1. Materials

C8-B4 cells, a mouse microglial cell line, were purchased from the American Type Culture Collection (Rockville, MD, USA; ATCC^®^, CRL-2540™). Opioid receptor agonists (DAMGO, DADLE, and U-50488), and lipopolysaccharide (LPS; *Escherichia coli* 055:B5), were purchased from Sigma-Aldrich (St. Louis, MO, USA). Cell culture media, Hanks’ balanced salt solution (HBBS), fetal bovine serum (FBS), and disposable plasticware were obtained from Thermo Fisher Scientific (Waltham, MA, USA). Protran (BA83 and BA85) nitrocellulose membranes were purchased from Schleicher & Schuell BioScience (Dassel, Germany). All other chemicals were from Merck KGaA (Darmstadt, Germany) and were of the highest available purity.

### 4.2. Cell Culture and Treatment

C8-B4 cells were cultured in Dulbecso’s modified Eagle’s medium supplemented with 10% FBS, 100 U/mL penicillin, 10 ug/mL streptomycin, and 25 ug/mL amphotericin B at 37 °C in a 5% CO_2_ incubator. In all experiments, cells were treated with various concentrations (0.01–1 μM) of selected opioid ligands (DAMGO, DADLE, and U-50488) for 1 h and then incubated in the presence of LPS (1 μg/mL) for an additional 24 h. Control (unstimulated) cells were treated with phosphate-buffered saline (PBS).

### 4.3. Cell Viability Assay

Cell viability was determined by MTT assay. C8-B4 microglial cells were seeded in 96-well plates at a density of 4 × 104 cells per well and incubated in a culture medium to allow cell adhesion. After pretreatment of the cells with opioid ligands for 24 h and subsequent incubation in the presence of LPS (1 μg/mL), 3-(4,5-dimethyltiazol-3-yl)-2,5-diphenylterazolium bromide (12 nmM) was added to each well, and the cells were incubated at 37 °C for 4 h. The culture medium was discarded, and 50 μL 0.1 M HCl in DMSO was added to dissolve the MTT-formazan crystals. Absorbance was measured at 570 nm compared to blank using a microplate reader (BioTek Synergy HT, Winooski, VA, USA).

### 4.4. Cytotoxicity Assay

Cytotoxicity was assessed using the Cytotoxicity Detection KitPLUS LDH (#04744926001; Merck) based on the determination of lactate dehydrogenase (LDH) activity released from the cytosol of damaged cells. The extent of cytotoxicity was determined in C8-B4 cells treated with opioid ligands and with or without LPS, as previously described [22].

### 4.5. Measurement of Intracellular ATP Content

Intracellular ATP content was determined by the ATP assay kit (ATP Bioluminescence Assay Kit CLS II; Merck) according to the manufacturer’s instructions, with minor adjustments. Cells were harvested by trypsinization and washed twice with PBS. Samples of 105 cells were centrifuged (800× *g*, 10 min), and pelleted cells were lysed by reconstitution in TE solution (100 mM Tris, 4 mM EDTA; pH 7.75) and heated at 95 °C for 7 min. Each sample was then mixed with luciferase reagent in a white 96-well plate and read in luminescence mode using a BioTek Synergy HT microplate reader.

### 4.6. Glucose Uptake Assay

Glucose uptake was determined by the Glucose Uptake-Glo Assay (Promega, Madison, WI, USA) according to the manufacturer’s recommendations. After treatment with OR agonists and LPS, cells were rinsed in warmed PBS, trypsinized, and placed in 50 μL PBS in white-bottomed 96-well plates, and 2-deoxyglucose (1 mM) was added for 20 min. After completion of the reaction and neutralization, 2-deoxyglucose-6-phosphate detection reagent was added, and luminescence was recorded for 30 min using a BioTek Synergy HT microplate reader.

### 4.7. Reduced Glutathione Assay

Reduced glutathione (GSH) levels were determined using the reduced glutathione assay kit (ab239709, Abcam, Cambridge, UK). C8-B4 cells grown in 12-well plates were treated with opioid ligands for 24 h and then incubated with and without LPS. Cells were then lysed by the addition of the lysis buffer (included in the kit) and centrifuged at 14,000× *g* for 10 min. GSH in the supernatant reacted with DTNB to generate yellow 2-nitro-5-thiobenzoic acid, the absorbance of which was measured using a BioTek Synergy HT microplate reader.

### 4.8. NADPH Assay

NADPH was measured using the NADPH assay kit (ab186031, Abcam, Cambridge, UK). C8-B4 cells grown in 12-well plates were treated with opioid ligands for 1 h and then incubated with and without LPS. Cells were then lysed by the addition of lysis buffer (included in the kit), sonicated, and centrifuged at 14,000× *g* for 15 min at 4 °C. NADPH in the supernatant was detected by the addition of the NADPH probe. The absorbance of the sample was measured using a BioTek Synergy HT microplate reader.

### 4.9. Mitochondrial Superoxide Assay

For flow cytometric detection of mitochondrial superoxide, C8-B4 cells were stained with MitoSOX as previously described [66], followed by flow cytometric analysis. Cells seeded in 24-well plates at a density of 105 cells/well were treated with opioid ligands and then with and without LPS as described above. Cells were then incubated with MitoSOX (5 μg/mL) for 30 min in the dark, washed twice with PBS, and harvested by trypsinization. Finally, fluorescence was determined using a BD LSR flow cytometer BD Bioscience and data analysis was performed using Kaluza 2.1 software.

### 4.10. Intracellular ROS Measurement

Intracellular ROS were assessed with 2′,7′-dihydrofluorescein diacetate (Sigma-Aldrich). C8-B4 cells grown in 12-well plates were pretreated with opioid ligands or LPS or H_2_O_2_ (50 nM), as indicated above. Cells were then incubated with DCFH-DA (10 μM) for 30 min. After washing with medium (once) and with PBS (twice), cells were observed with a fluorescence microscope (AIF, Arsenal, Czech Republic) at Ex/Em 495 nm/527 nm, and fluorescence intensity was assessed and corrected for the number of cells per image using ImageJ 1.53u software.

### 4.11. High-Resolution Respirometry

Cellular oxygen consumption rate was measured by high-resolution respirometry using a two-channel titration injection respirometer Oxygraph-2k (Oroboros Instruments, Innsbruck, Austria) in standard configuration, with 2.1 mL final volume of treated and control cells in both chambers at 37 °C and 750 rpm. The protocol consisted of sequential application of oligomycin (Sigma), carbonyl cynide-4-trifluromethoxyphenylhydrazone (FCCP, Sigma), and antimycin A, as previously reported [67]. For all experiments, 5 million cells per chamber were used, and data were expressed in pmol O_2_·s-1 per cell. The Oroboros DatLab 7.4.0.4 software (Oroboros Instruments) was used for data analysis.

### 4.12. Western Blot Analysis

Western blot experiments were performed as previously described. Cells were lysed in RIPA lysis buffer containing protease inhibitors (cOmplete protease inhibitor cocktail, Sigma-Aldrich). The lysates (equal amounts of protein) and Laemmli sample buffer were mixed and boiled for 2 min before application to the gel (Bio-Rad, Hercules, CA, USA). Electrophoresis was performed at constant voltage (200 V) for approximately 60 min until the dye front reached the end of the 50-mm gel. Then, the separated proteins were transferred to nitrocellulose membranes with a pore size of 0.45 µm or 0.2 μm. The membranes were blocked with 5% skim milk in TBS-T buffer (10 mM Tris, 150 mM NaCl, 1% Tween 20; pH 8.0) for 1 h at room temperature and then incubated with primary antibodies overnight at 4 °C. The primary antibodies used in this study are listed in the Supplementary Materials (Supplementary Table S1). After rinsing with TBS-T buffer, blots were incubated with appropriate horseradish peroxidase-linked secondary antibodies (anti-mouse/anti-rabbit/anti-goat immunoglobulin G) for 1 h at room temperature. After washing off the unbound probes, the membrane was incubated with Super Signal chemiluminescent substrates (Pierce Biotechnology, Rockford, IL, USA) for 1 min. Blots were scanned and quantitatively analyzed using ImageJ software. Immunochemical signal intensities were normalized to total protein determined by Ponceau S staining, and results were expressed as fold change relative to control.

### 4.13. Statistical Analysis

All experiments were performed in at least three independent biological replicates. Statistical analyzes were performed using GraphPad Prism software version 8.0 (GraphPad Software, San Diego, CA, USA). All data were expressed as mean ± SEM (standard error of the mean). Differences between groups were analyzed by one-way analysis ANOVA followed by Tukey’s multiple comparison post hoc test. *p* values of less than 0.05 were considered statistically significant.

## Data Availability

All the data supporting the findings of this study are available within the paper.

## Supplementary Materials

The supporting information can be downloaded at: https://www.mdpi.com/article/10.3390/ijms241311089/s1.
