# Supplementary material for: Opioids Alleviate Oxidative Stress via the Nrf2/HO-1 Pathway in LPS-Stimulated Microglia
Source: Int J Mol Sci. 2023 Jul 4;24(13):11089. doi: 10.3390/ijms241311089 (PMC10342087; doi:10.3390/ijms241311089)
Supplement: Supplementary file 1 [file ijms-24-11089-s001.zip › ijms-2482874-supplementary.pdf]

Supplementary. Fig. S1. Effects of LPS and opioid receptor agonists on cell viability, LDH release, ATP content and GLUTs expression. C8-B4 cells were either untreated (control, C) or treated with LPS (1 μg/ml, L) or different concentrations (0.01-1 μM) of DAMGO, DADLE and U-50488 for 24 h before assessment of cell viability (A,B,C), cytotoxicity (D,E,F), ATP (G), NADPH (H), GLUT2 (I) or GLUT4 (J). Data are presented as means ± SEM of three independent experiments performed in triplicate (**p<0.01; ***p<0.001; ****p<0.0001 compared with control group).

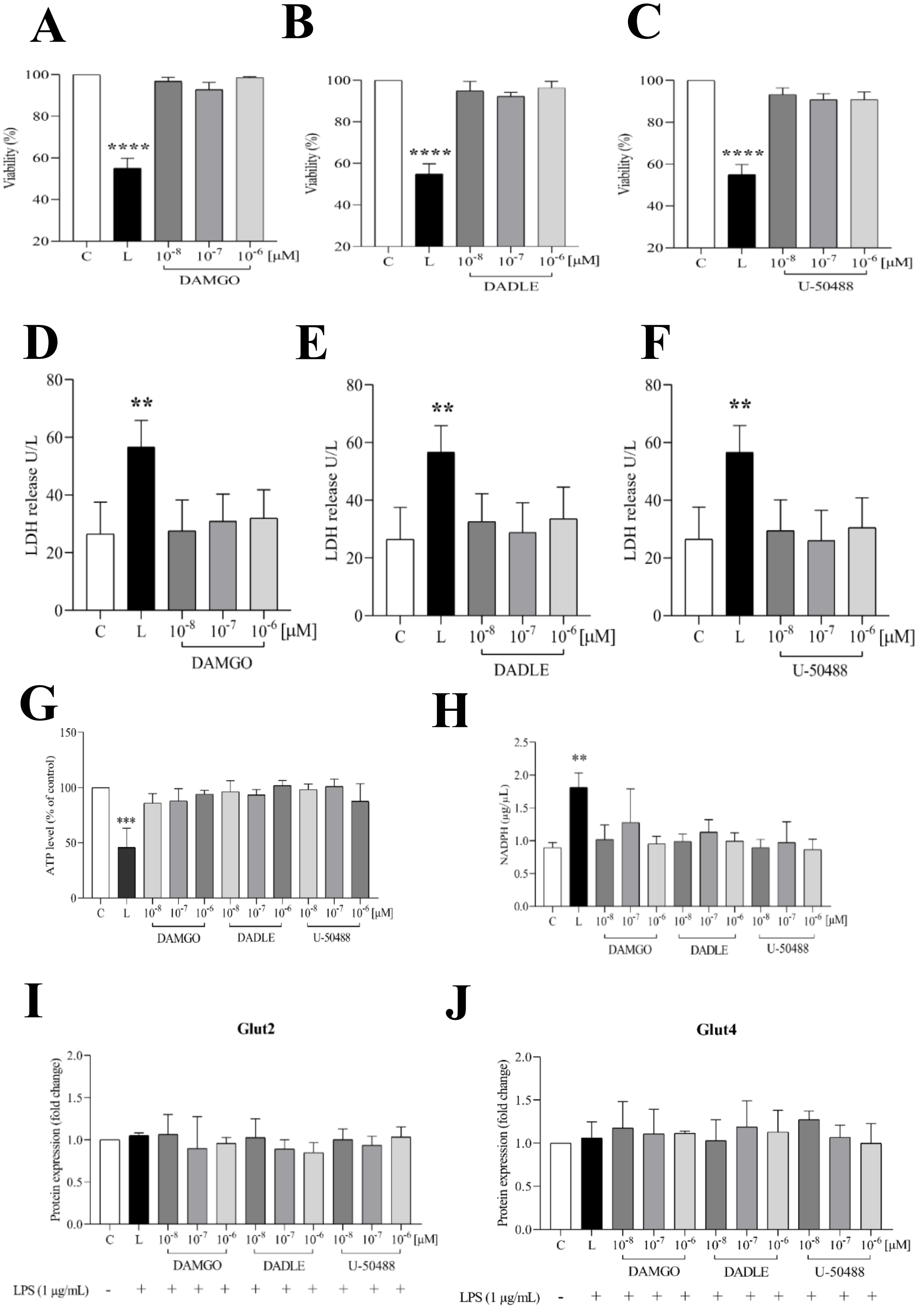

Supplementary. Fig. S2. Effects of LPS and opioid agonists on the expression of selected antioxidant enzymes and HO-1/Nrf2. C8-B4 cells were either untreated (control, C) or treated with LPS (1 μg/ml, L) or different concentrations (0.01-1 μM) of DAMGO, DADLE and U-50488 for 24 h. The levels of selected markers were determined western blot analysis (A) and the relative levels of HO-1 (B), Nrf-2 (C), SOD2 (D), SOD3 (E), Catalase (F) or Gpx1/2 (G) were expressed as fold changes compared with control. Data are presented as means ± SEM of two independent experiments (n=2) (*p<0.05 compared with control group).

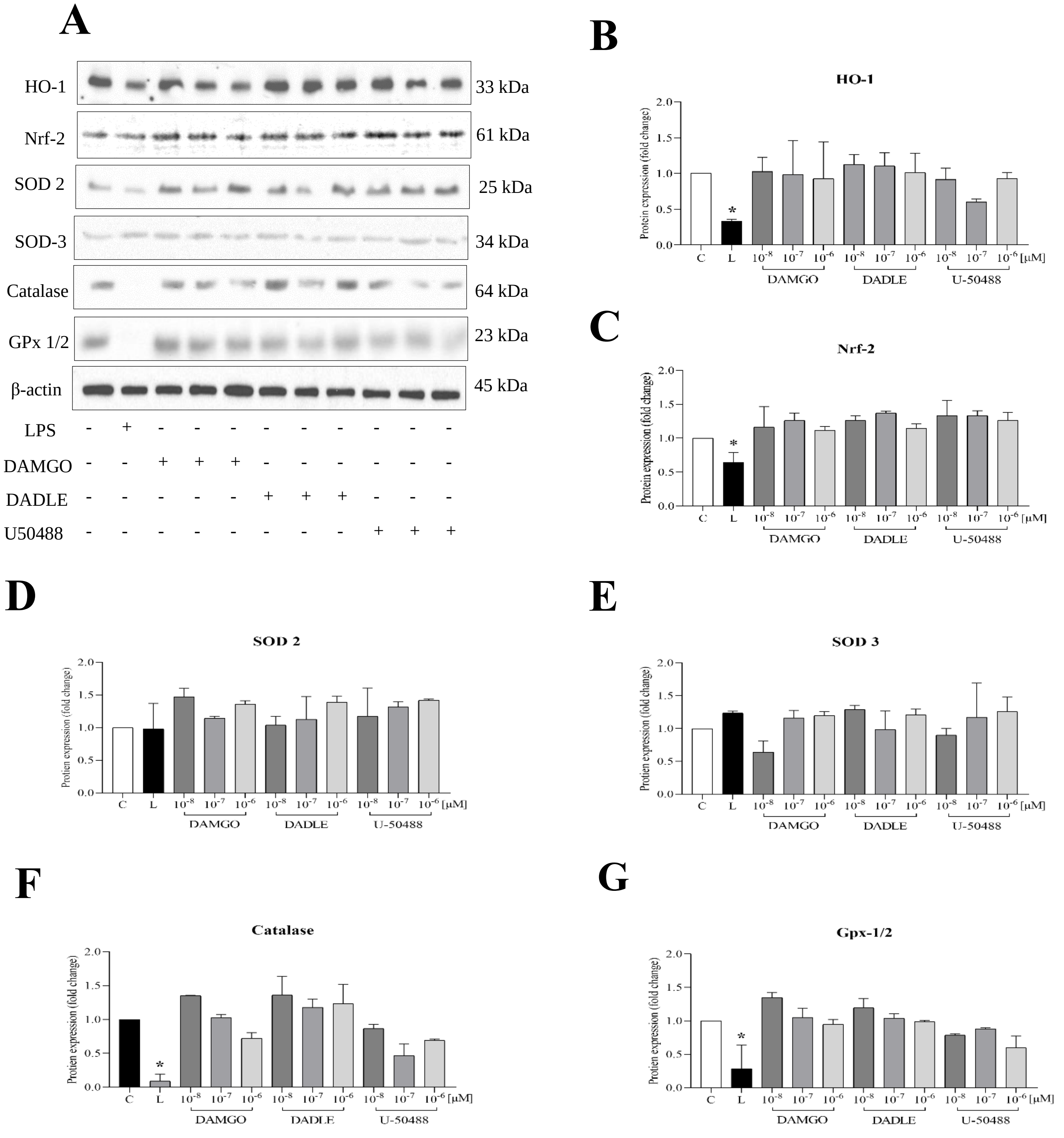

Supplementary. Fig. S3. Effect of LPS and opioid agonists on mitochondrial respiration and ROS. C8-B4 cells were either untreated (control) or pretreated with opioid ligands (DAMGO, DADLE, U-50488) at a concentration of 1 μM for 1 h and then incubated in the presence of LPS (1 μg/mL, L) for 24 hours. Five million cells were used for each experiment, and the oxygen consumption rate was determined under different experimental conditions as indicated in the Methods. (A). The original recordings of respiration from DatLab. Mitochondrial ROS were determined by flow cytometric analysis using the MitoSOX Red probe. (B). Representative flow cytometric histogram of each group. Gray, ungated; green, control; red, LPS; descending blue shades, DAMGO (0.01, 0.1, and 1 μM); descending pink shades, DADLE (0.01, 0.1, and 1 μM); descending orange shades, U-50488 (0.01, 0.1, and, 1 μM).

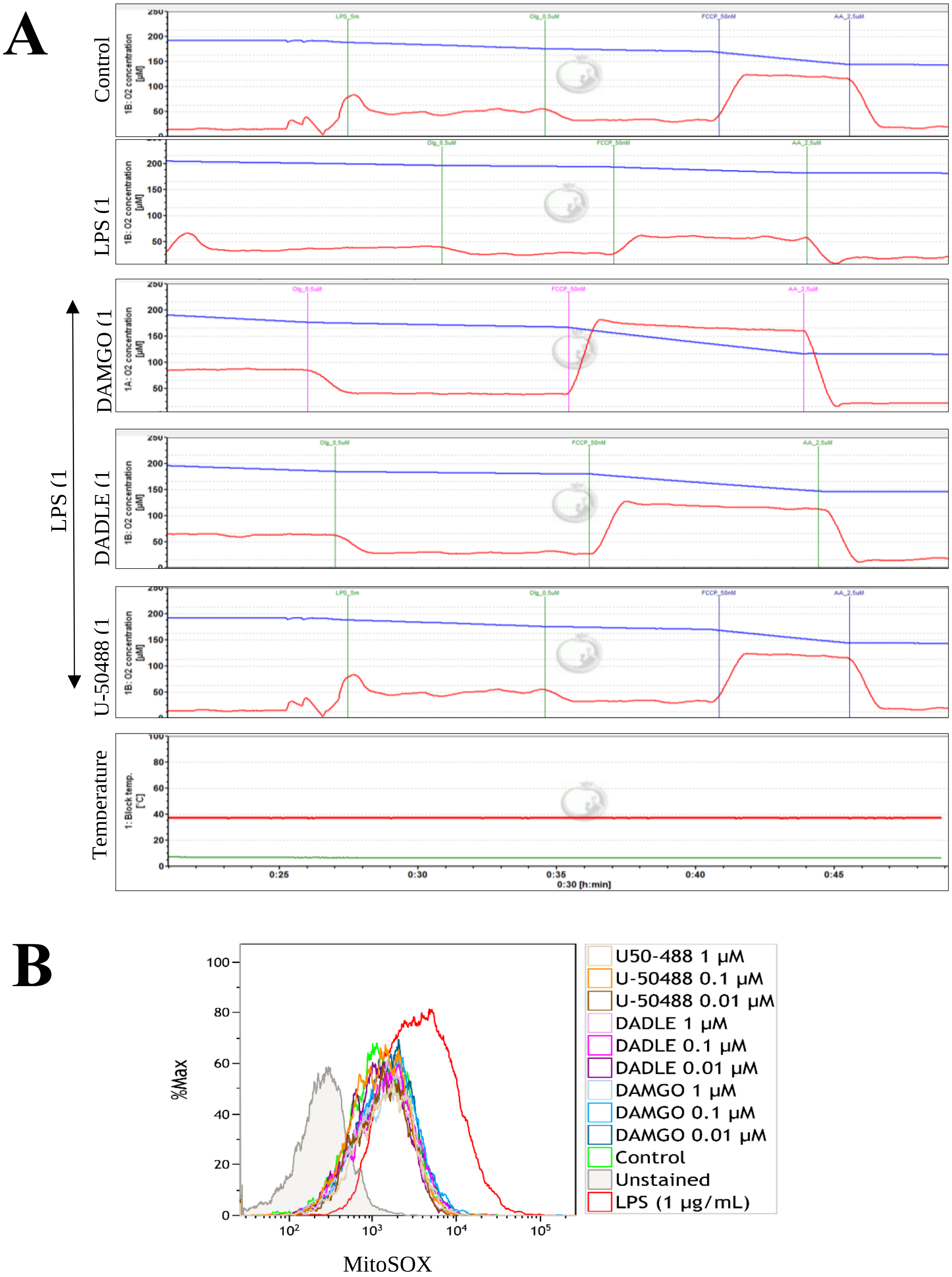

Suppl. table. S1. List of antibodies

No	Antibodies	Provider	Catalog number
1	anti-catalase	Santa Cruz Biotechnology, Inc. (Dallas, TX, USA).	sc-271803
2	anti-GPx-1/2	Santa Cruz Biotechnology, Inc. (Dallas, TX, USA).	sc-133160
3	anti-HO-1	Santa Cruz Biotechnology, Inc. (Dallas, TX, USA).	sc-136960
4	anti-Nrf2	Santa Cruz Biotechnology, Inc. (Dallas, TX, USA).	sc-722
5	anti-SOD2	Santa Cruz Biotechnology, Inc. (Dallas, TX, USA).	sc-30080
6	anti-SOD3	Santa Cruz Biotechnology, Inc. (Dallas, TX, USA).	sc-32222
7	Anti-Glucose Transporter GLUT1	Abcam (Cambridge, United Kingdom)	ab-652
8	Anti-Glucose Transporter GLUT2	Bioss (Boston, MA)	Bs-0351
9	Anti-Glucose Transporter GLUT3	Abcam (Cambridge, United Kingdom)	ab-15311
10	Anti-Glucose Transporter GLUT4	Abcam (Cambridge, United Kingdom)	ab-654
11	Beta actin	Santa Cruz Biotechnology, Inc. (Dallas, TX, USA).	sc-47778
